# Omega 3 fatty acids stimulate thermogenesis during torpor in the Arctic Ground Squirrel

**DOI:** 10.1038/s41598-020-78763-8

**Published:** 2021-01-14

**Authors:** S. A. Rice, M. Mikes, D. Bibus, E. Berdyshev, J. A. Reisz, S. Gehrke, I. Bronova, A. D’Alessandro, K. L. Drew

**Affiliations:** 1 Department of Chemistry and Biochemistry, Center of Transformative Research in Metabolism, University of Alaska Fairbanks, Fairbanks, AK USA; 2 Institute of Arctic Biology, Center of Transformative Research in Metabolism, University of Alaska Fairbanks, Fairbanks, AK USA; 3 Lipid Technologies, LLC, Austin, MN USA; 4 National Jewish Health, Denver, CO USA; 5 Department of Biochemistry and Molecular Genetics, University of Colorado Anschutz Medical Campus, Aurora, CO USA

**Keywords:** Metabolism, Lipids, Animal physiology

## Abstract

Omega 3 polyunsaturated fatty acids (PUFAs) influence metabolism and thermogenesis in non-hibernators. How omega 3 PUFAs influence Arctic Ground Squirrels (AGS) during hibernation is unknown. Prior to hibernation we fed AGS chow composed of an omega 6:3 ratio approximately 1:1 (high in omega 3 PUFA, termed Balanced Diet), or an omega 6:3 ratio of 5:1 (Standard Rodent Chow), and measured the influence of diet on core body temperature (T_b_), brown adipose tissue (BAT) mass, fatty acid profiles of BAT, white adipose tissue (WAT) and plasma as well as hypothalamic endocannabinoid and endocannabinoid-like bioactive fatty acid amides during hibernation. Results show feeding a diet high in omega 3 PUFAs, with a more balanced omega 6:3 ratio, increases AGS T_b_ in torpor. We found the diet-induced increase in T_b_ during torpor is most easily explained by an increase in the mass of BAT deposits of Balanced Diet AGS. The increase in BAT mass is associated with elevated levels of metabolites DHA and EPA in tissue and plasma suggesting that these omega 3 PUFAs may play a role in thermogenesis during torpor. While we did not observe diet-induced change in endocannabinoids, we do report altered hypothalamic levels of some endocannabinoids, and endocannabinoid-like compounds, during hibernation.

## Introduction

Hibernation is composed of periods of radically reduced whole body metabolism and core body temperature (T_b_) that last for weeks (torpor bouts) which are regularly interrupted by interbout arousals (IBA) when metabolism and T_b_ briefly return to euthermic levels^1,2^. AGS fuel the majority of their whole-body metabolism from fat stores through the hibernation season while fasting^2,3^. It is well-documented that specific types of lipids can regulate hibernation; the omega 6 polyunsaturated fatty acid (PUFA) linoleic acid (LA) has been known for decades to influence T_b_ and duration of torpor bouts in multiple species^4–10^. Fewer studies have investigated omega 3 PUFAs with conflicting results showing feeding omega 3 PUFAs prior to hibernation (1) inhibited hibernation in marmots^11^, (2) delayed seasonal hibernation onset in garden dormice^12^ or (3) had no influence on hibernation in ground squirrels^7^.

Recent studies in non-hibernating animals show stimulation of brown adipose tissue (BAT) and metabolism from omega 3 PUFAs^13,14^. Omega 3 PUFA action on thermogenesis may arise from beiging of white adipose tissue (WAT), enhancement of mitochondrial respiration in BAT or modulating miRNAs through free fatty acid receptors^13,15–17^. Thermogenesis, as well as hibernation, are also both regulated centrally in part by the preoptic area of the hypothalamus^18–23^. Our laboratory previously highlighted the role of the hypothalamus in increasing thermogenic capacity through the hypothalamic-pituitary-thyroid (HPT) axis in hibernation^24^. Endocannabinoid signaling also modulates thermogenesis through actions on the hypothalamus^25^. PUFAs are the parent compounds of multiple endocannabinoid and endocannabinoid-like molecules, known to modulate metabolism, thermogenesis, circannual rhythms, neurotransmission, the immune system and satiation^26–34^. Further, hypothalamic endocannabinoid levels are maintained in hibernation compared to summer in marmots^35^. Major endocannabinoids, such as 2-arachidonoylglycerol (2-AG) and anandamide (AEA), are derived from the omega 6 PUFA arachidonic acid, and other N-acylethanolamides (NAEs) are derived from multiple types of other fatty acids, such as omega 3 PUFAs or saturated fatty acids^36,37^. Some studies have found that altering PUFAs and other fats in feed can influence bioactive NAEs and endocannabinoid levels in the brain^34,36,38^.

Given omega 3 PUFAs are prominent in some free-ranging hibernator diets^39,40^, we hypothesized omega 3 PUFAs influence hibernation. Specifically, we hypothesized that the Balanced Diet would increase thermogenesis and that hypothalamic bioactive endocannabinoids, such as 2-AG and AEA, are influenced by diet. To test this hypothesis, we fed either Balanced Diet (LabDiet, 9GU5, high in omega 3 PUFAs) or a control diet, Standard Rodent Chow (Mazuri #5663, high in omega 6 PUFAs), to juvenile AGS after capture and quarantine in July until animals hibernated and recorded T_b_ throughout hibernation. We then measured hypothalamic NAEs, plasma, WAT and BAT fatty acids and BAT tissue mass during torpor and IBA in December. Our findings support a role for omega 3 PUFAs in increasing AGS T_b_ in torpor and BAT mass during hibernation.

## Results

### Feeding a diet high in omega 3 PUFAs with a more balanced omega 6:3 ratio increased core body temperature in torpor

Previous work in non-hibernating mammals indicate omega 3 PUFAs can influence metabolism and thermogenesis^15,16^, but hibernation studies have found conflicting evidence^7,11,12^. Therefore, the first goal was to measure the influence of omega 3 PUFAs on Arctic Ground Squirrels (AGS) core body temperature (T_b_) during hibernation. Feeding the Balanced Diet, which is heavy in alpha-linolenic acid (ALA), increased T_b_ in torpid AGS (Table 1a, p = 0.020, t-test). Torpor bout length and T_b_ during interbout arousal were not influenced by diet (Table 1a). Balanced Diet (Lab Diet 9GU5) composition predominately differed from Standard Rodent Chow (Mazuri, #5663) by increased ALA and decreased linoleic acid (LA) (Table 1b).

**Table 1:** Balanced Diet increases core body temperature in torpid AGS**.**

(a)	Balanced diet ω 6:3	Standard Rodent Chow
Core body temperature (°C) during Torpor	3.3 (± 0.1)*, n = 9	2.8 (± 0.1), n = 8
Core body temperature (°C) during Arousal	35.6 (± 0.1), n = 9	35.5 (± 0.1), n = 8
First Torpor bout length (h)	136.2 (± 20.4) n = 9	172.1 (± 29.0) n = 8
Second Torpor bout length (h)	237.7 (± 14.5) n = 9	237.4 (± 24.8) n = 8
Third Torpor bout length (h)	315.8 (± 18.4) n = 9	303.6 (± 28.6) n = 8
Fourth Torpor bout length (h)	351.7 (± 15.6) n = 9	370.6 (± 26.7) n = 7
Fifth Torpor bout length (h)	375.8 (± 19.5) n = 8	388.3 (± 23.6) n = 7

(b)	Balanced diet ω 6:3	Standard Rodent Chow
**Percent total fatty acid**		
Palmitic acid (16:0)	13.99	12.49
Palmitoleic acid (16:1ω7)	1.19	0.24
Stearic acid (18:0)	4.89	3.44
Oleic acid (18:1ω9)	24.67	20.96
Linoleic acid (18:2ω6)	29.58	50.50
Alpha linolenic acid (18:3ω3)	19.81	10.04
Arachidonic acid (20:4ω6)	0.20	0.02
EPA (20:5ω3)	0.69	0.04
DHA (22:6ω3)	0.96	0.11
Tot.ω3	21.81	10.24
Tot.ω6	30.09	50.67
Tot.ω9	24.77	21.01
Ratio ω6/ω3	1.38	4.95
**Concentration (mg/100 g chow)**		
Total fat	7326.34	7484.87
Total PUFA	3620.8	4342.3

(a) Hibernating core body temperature is significantly higher in Balanced Diet fed AGS (*p = 0.038, t-test, n = 8–9), but diet did not influence arousal core body temperature or torpor bout length. Data shown are mean ± SEM. (b) Fatty acid composition of Balanced Diet (Lab Diet, 9GU5) and Standard Rodent Chow (Mazuri, #5663). Data shown are percent of total fatty acid, except total fat and total PUFA are mg/100 g chow. Full fatty acid composition is listed in Supplemental Table 7.

### Feeding a diet high in omega 3 PUFAs altered fatty acid composition of brown adipose tissue, white adipose tissue and plasma in hibernators

**Figure 1:**
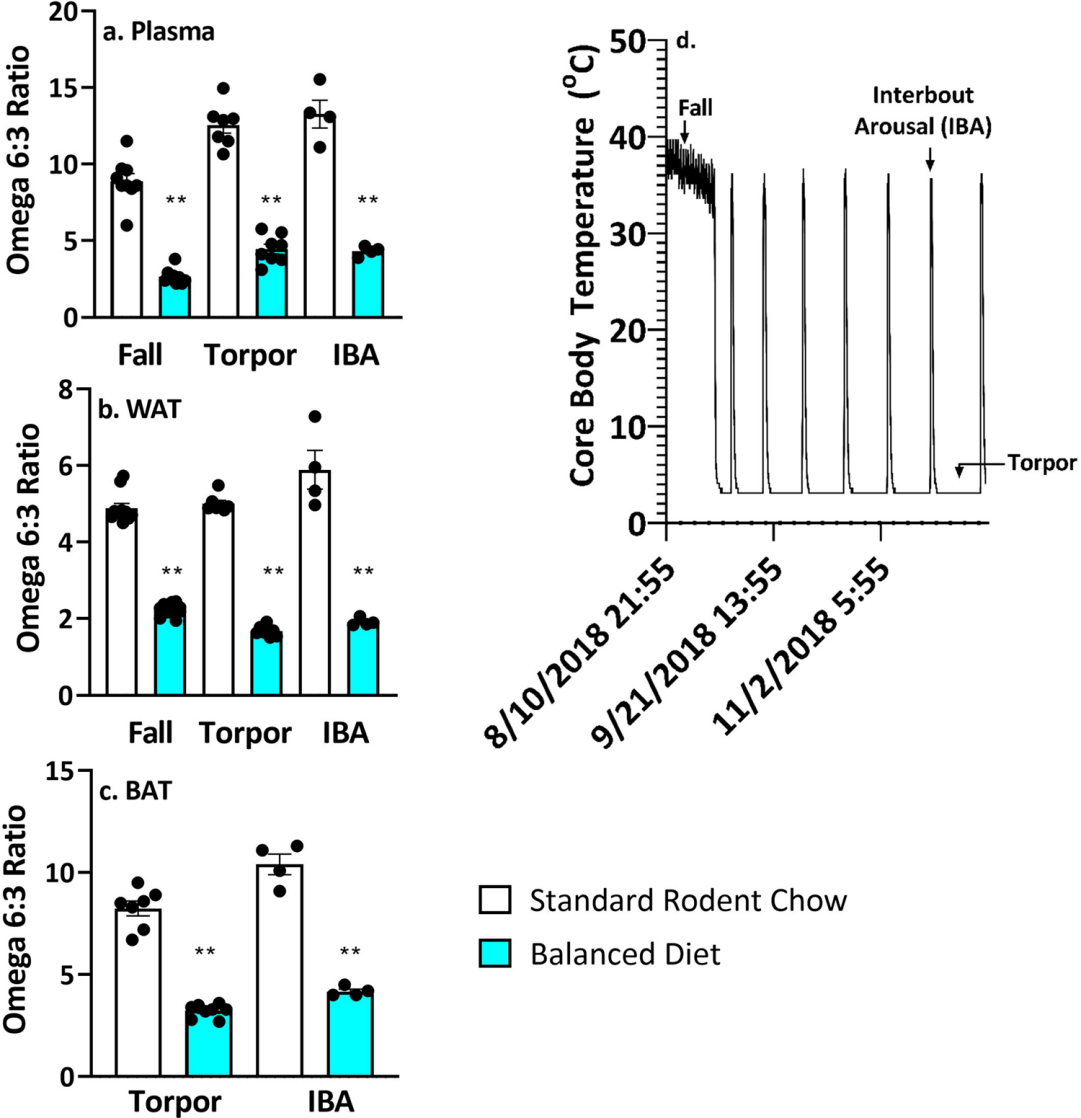
Omega 6:3 ratio is lower in plasma, WAT and BAT of Balanced Diet AGS. (**a**) Plasma, **p < 0.001, two tailed t-test, fall n = 9, torpor n = 7–8, IBA n = 4. (**b**) WAT, **p < 0.001, two-tailed t-test, fall n = 11–12, torpor n = 7–8, IBA n = 4. (**c**) BAT, **p < 0.001, two tailed t-test, torpor n = 7–8, IBA n = 4. (**d**) An example of time points when AGS were sampled over the hibernation season. Data shown are mean ± SEM. Black dots are individual data points.

AGS fast and rely on fat stores during hibernation. We asked how feeding the Balanced Diet prior to hibernation influenced fatty acid content in hibernating fat deposits (visceral white adipose tissue, WAT), brown adipose tissue (BAT, a major thermogenic tissue) and plasma. Feeding the Balanced Diet significantly decreased the omega 6:3 ratio in BAT, WAT and plasma in hibernating animals during torpor and IBA compared to the Standard Rodent Chow (Fig. 1, p < 0.05, two-tailed t-test).

**Figure 2:**
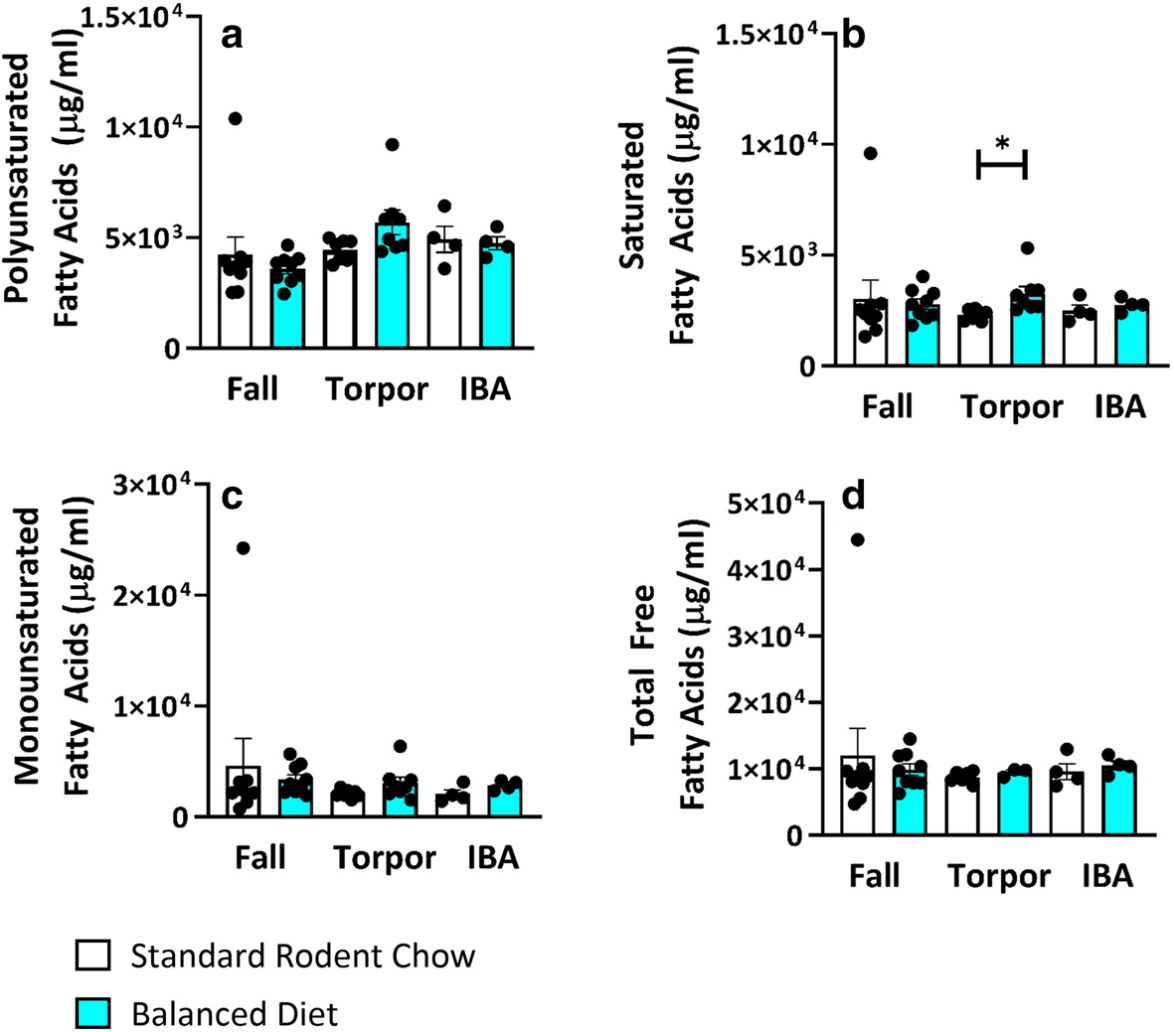
In plasma, Balanced Diet does not increase Total PUFAs (**a**), MUFAs (**c**) or Free Fatty Acids (**d**). Balanced Diet does increase saturated fatty acids during torpor (**b**). *p < 0.05, two-tailed t-test, FDR corrected. Data shown are mean ± SEM. Black dots are individual data points. Fall n = 9, Torpor n = 7–8, IBA n = 4 per diet.

Balanced Diet did not significantly alter total polyunsaturated fatty acids (PUFA), monounsaturated fatty acids (MUFA) or total free fatty acids (FFA) in AGS plasma compared to the control diet animals during the hibernation season (Fig. 2). Balanced Diet did, however, increase saturated fatty acids (SFA) in torpor compared to control AGS (Fig. 2, p < 0.05, two-tailed t-test, FDR corrected).

### Docosahexaenoic acid (DHA) and eicosapentaenoic acid (EPA) were elevated in balanced diet plasma while omega 6 PUFAs were unchanged

**Figure 3:**
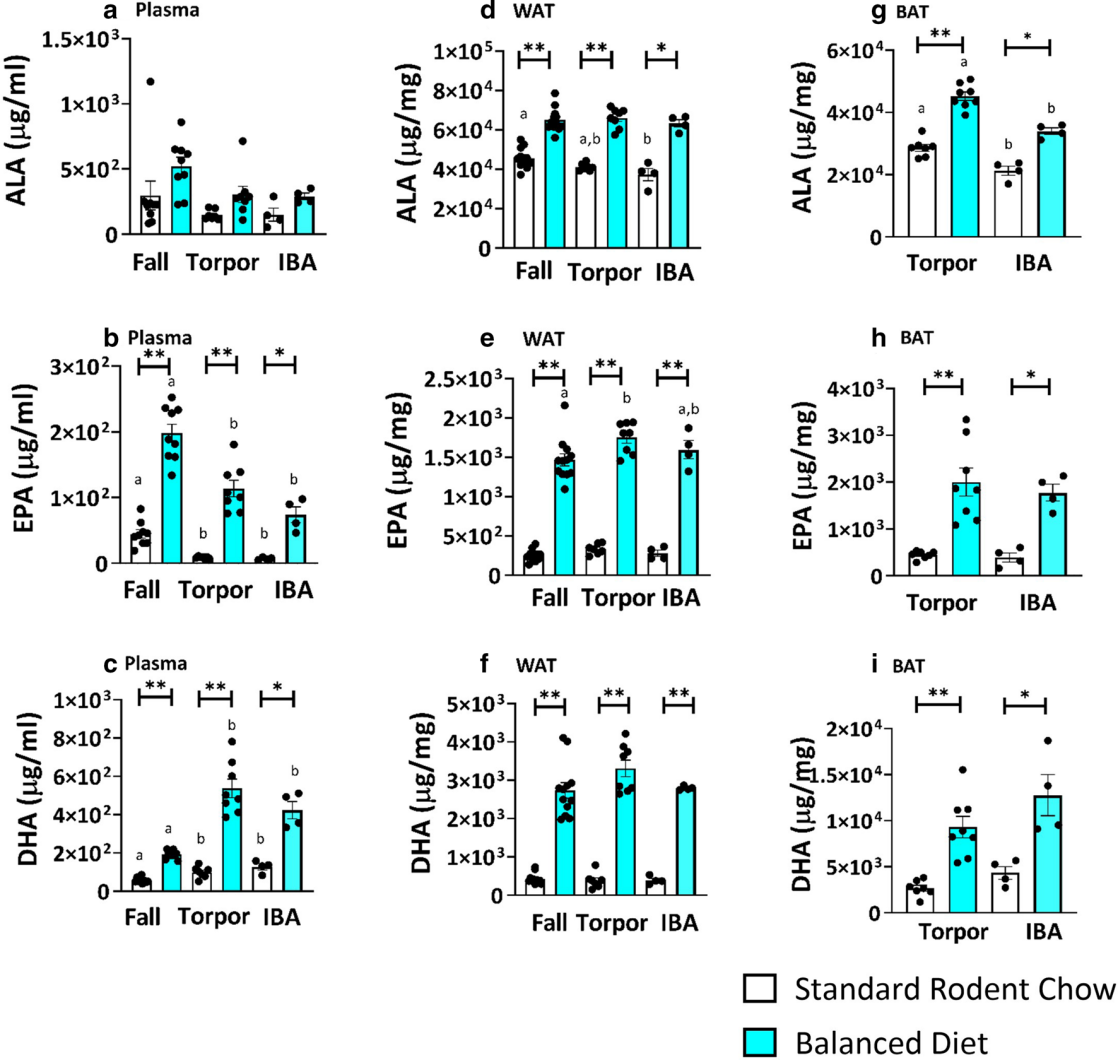
Feeding Balanced Diet leads to high omega 3 PUFAs in plasma and adipose tissue. (**a**) Plasma DHA and EPA are increased in Balanced Diet (BD) at all time points compared to Standard Rodent Chow (SRC) (*p < 0.05, **p < 0.001, t-test, FDR corrected, fall n = 9, torpor n = 7–8, IBA n = 4). Seasonal comparisons within the same diet found DHA was highest in torpor and IBA (post hoc Tukey, letters (a or b) within a given diet signify differences between phenotypic states within that diet). (**b**) WAT ALA, EPA and DHA are significantly increased in BD AGS compared to SRC AGS in all time points (*p < 0.05, **p < 0.001, t-test, FDR corrected, fall n = 11–12, torpor = 7–8, IBA = 4). (**c**) BAT ALA, EPA and DHA are significantly higher in BD AGS compared to SRC AGS in torpor and IBA (*p < 0.05, **p < 0.001, t-test, FDR corrected, torpor = 7–8, IBA = 4). Comparisons between torpor and IBA within the same diet found ALA decreased in IBA compared to torpor (t-test, FDR corrected, letters (a or b) within a given diet signify differences between phenotypic states within that diet). Fall BAT was not sampled. Data shown are mean ± SEM. Black dots are individual data points.

As DHA and EPA activate thermogenic mechanisms in non-hibernators^15,16^, we were interested in their presence in circulation and tissues during hibernation. AGS rely principally on fat stores during hibernation while fasting. In all tissues and plasma studied, feeding the Balanced Diet significantly increased DHA and EPA levels compared to the control diet (Fig. 3, two-tailed t-test, FDR corrected). While plasma alpha-linolenic acid (ALA) did not increase, Balanced Diet increased ALA in WAT and BAT (Fig. 3a,d,g, p < 0.05, two-tailed t-test, FDR corrected). During the hibernation season (torpor and IBA) plasma DHA was more abundant than in the fall regardless of diet (Fig. 3c, p < 0.05 one-way ANOVA, post hoc Tukey). DHA did not show the same seasonal increase in WAT (Fig. 3b) and fall samples were not available for BAT (Fig. 3g–i). BAT was the only tissue, however, where one of the omega-3 PUFAs (ALA) decreased during IBA (Fig. 3g). In BAT, ALA was significantly less during IBA than during torpor (Fig. 3g, p < 0.05, t-test, FDR corrected).

**Figure 4:**
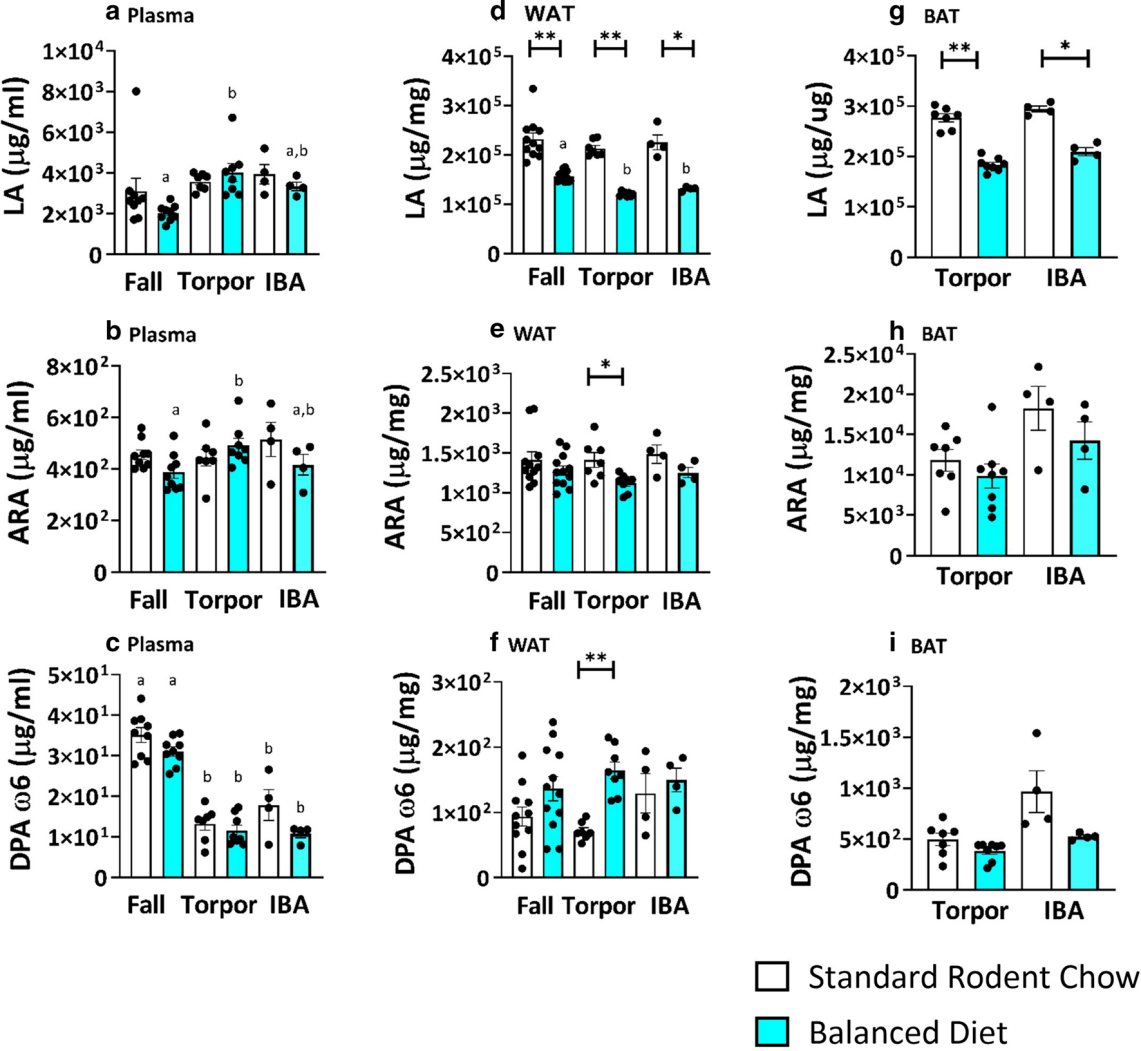
Balanced Diet does not influence omega 6 PUFAs in plasma, but diet influences LA in WAT and BAT. (**a**) Plasma LA, ARA and DPA w3 does not differ between Balanced Diet (BD) AGS in all time points compared to Standard Rodent Chow (SRC) AGS (t-test, FDR corrected, fall n = 9, torpor n = 7–8, IBA n = 4). Seasonal comparison within the same diet found plasma LA increases in torpor compared to fall BD AGS while DPA w6 decreases in torpor and IBA compared to fall in both diets (post hoc Tukey, letters (a or b) signify difference between season within the same diet group). (**b**) WAT LA is increased in SRC AGS compared to BD AGS in fall, torpor and IBA, while ARA is increased in torpid SRC animals and DPA w6 is increased in BD AGS in torpor (*p < 0.05, **p < 0.001, t-test, FDR corrected, fall n = 11–12, torpor = 7–8, IBA = 4). Seasonal comparison within the same diet found WAT LA decreases in torpor compared to fall BD AGS (post hoc Tukey, letters (a or b) signify difference between season within the same diet group) **c.** BAT LA is significantly higher in SRC AGS compared to BD AGS in torpor and IBA (*p < 0.05, **p < 0.001, t-test, FDR corrected, torpor = 7–8, IBA = 4). Data shown are mean ± SEM. Black dots are individual data points.

The Balanced Diet had a less consistent effect on plasma levels of omega 6 PUFAs than on levels of omega 3 PUFAs. In plasma, linoleic acid (LA), arachidonic acid (ARA) and docosapentaenoic acid (DPA ω6) did not differ between diets (Fig. 4a–c). In WAT and BAT, Balanced Diet decreased LA regardless of season or phenotypic state (Fig. 4d,g, p < 0.05, two-tailed t-test, FDR corrected). In WAT, ARA also decreased in Balanced Diet, but only during torpor (Fig. 4e, p < 0.05, two-tailed t-test, FDR corrected). This decrease in ARA occurred despite a tenfold higher amount of ARA in Balanced Diet chow compared to Standard Rodent Chow (Table 1b). BAT ARA and DPA ω6 did not differ between diets during hibernation (Fig. 4 h,i). By contrast, in WAT, Balanced Diet increased DPA ω6 during torpor (Fig. 4f, p < 0.05, two-tailed t-test, FDR corrected).

### Balanced diet increased axillar brown adipose tissue deposits during hibernation

**Figure 5:**
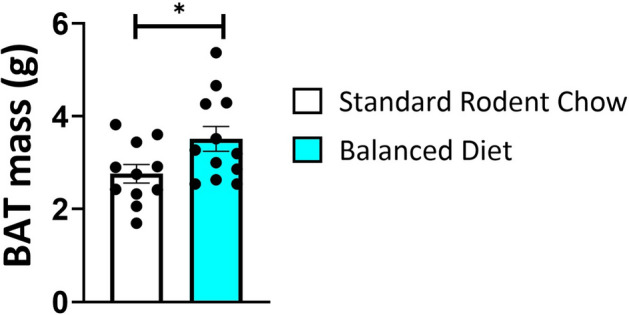
Brown Adipose Tissue (BAT) mass is increased in Balanced Diet AGS (*p = 0.038, n = 11 Standard Rodent Chow, n = 12 Balanced Diet, t-test). BAT mass was sampled during the hibernation season in December. Data shown are mean ± SEM. Black dots are individual data points.

As omega 3 PUFAs have been shown to induce BAT adipogenesis^15^, we investigated if feeding omega 3 PUFAs increased BAT mass during the hibernation season. AGS fed the Balanced Diet had significantly more BAT mass than AGS fed the Standard Rodent Chow when measured in December during hibernation tissue collection (Fig. 5, p = 0.038, t-test).

### Long chain PUFAs are produced during natural interbout arousal from torpor

**Figure 6:**
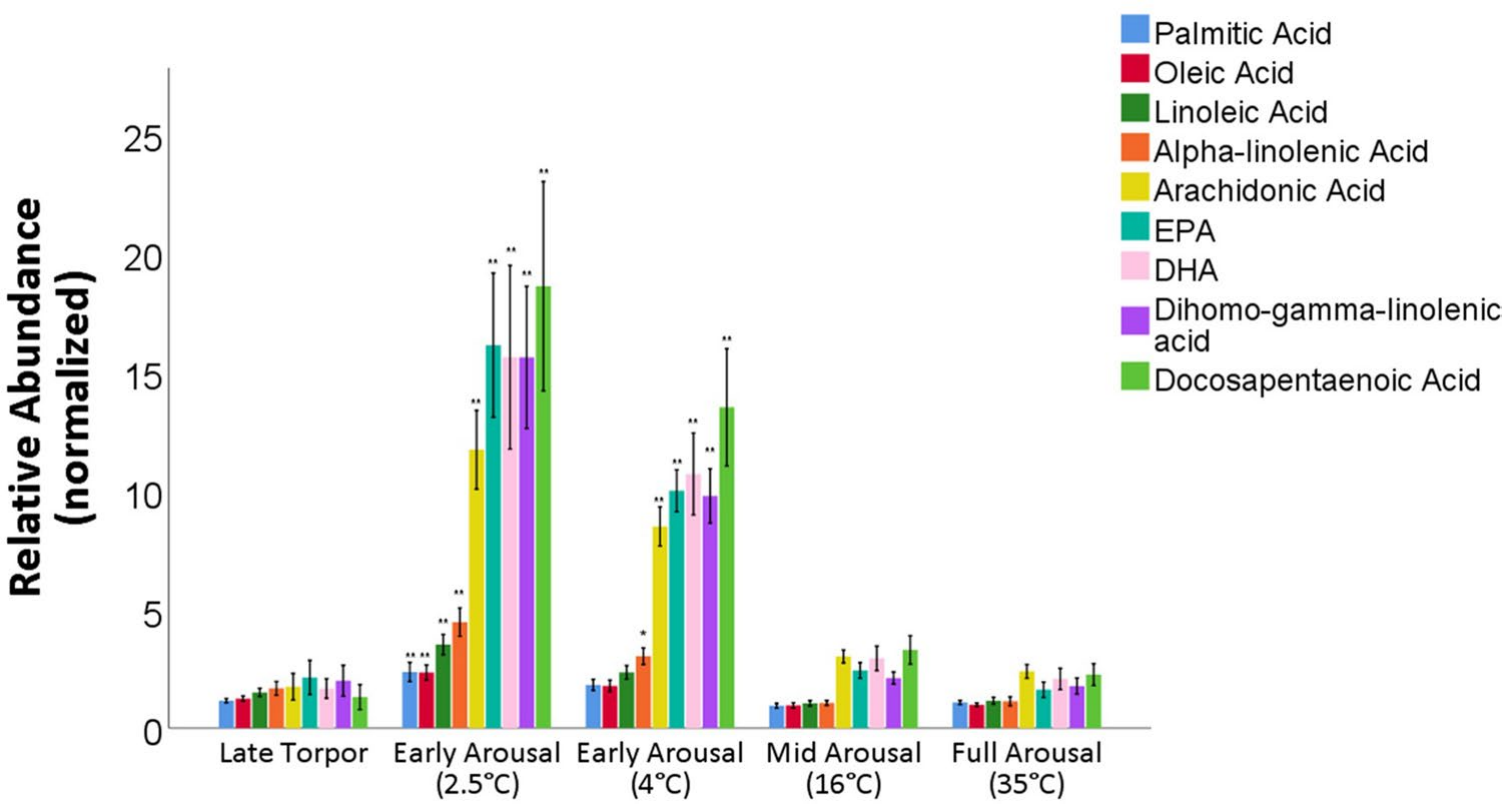
Plasma metabolomic analysis during natural arousal from torpor shows spike of PUFA metabolites during early arousal. Plasma Fatty Acids and PUFAs increase significantly during early natural arousal from torpor. Data shown are relative abundance normalized to plasma sample collected at entrance into torpor at a T_b_ of 11–12 °C. The x-axis are phases of rewarming in natural arousal from torpor. (*p < 0.05, **p < 0.001 vs late torpor, post hoc Tukey, n = 9). Measurements are from individual animals sampled over a single, undisturbed natural arousal fed Standard Rodent Chow. Blood was sampled at specific core body temperatures (T_b_) during arousal ranging from early arousal (2.5 °C) to full arousal (35 °C). Data shown as mean $\pm$ SEM.

We next asked how the energetically demanding process of arousal from torpor affected lipid plasma profiles. For this study, where we only had access to AGS fed Standard Rodent Chow, we found that long chain PUFAs increased during early interbout arousal (IBA) (Fig. 6). Early interbout arousal is characterized by high metabolic rate and BAT thermogenesis^41,42^. Saturated, monounsaturated and polyunsaturated fatty acids all significantly increased during early arousal (when T_b_ is approximately 2.5 °C) (Fig. 6, p < 0.05, ANOVA, post hoc Tukey, n = 9). Downstream metabolites of the omega 6 and 3 cascade, such as DHA, EPA, dihomo-gamma-LA, DPA and ARA increased approximately 5–8-fold compared to late torpor and remained elevated as the animals’ core body temperature rose to 4 °C (Fig. 6). Further, specific interbout arousal periods were separated into distinct metabolic phenotypes based on lipid profile, distinct from full interbout arousal as well as from torpor (Supp. Fig. 1a, partial least-squares discriminate analysis). Pathway enrichment analysis additionally supports that PUFA chain elongation and desaturation was significant during interbout arousal (Supp Fig. 1b, p < 0.0001, n = 9).

### Balanced diet did not alter major hypothalamic endocannabinoids

The endocannabinoid system modulates metabolism, thermogenesis and circannual rhythms within the hypothalamus^25,33^; endocannabinoids and bioactive NAEs derive from PUFAs and fatty acids^26,37^. Given that previous studies have found dietary fats could sometimes alter brain NAE composition^34,36^, we next asked if an increase in omega 3 PUFAs through diet influenced hypothalamic endocannabinoid levels in hibernation. The majority of endocannabinoids and NAEs did not differ between the two diets in the hypothalamus during torpor, but some saturated NAEs did differ. During torpor 14:0 NAE (Supp. Table 1, p = 0.008, two-tailed t-test, FDR corrected) was significantly higher in Balanced Diet fed AGS compared to Standard Rodent Chow fed AGS while 22:0 NAE was higher in Standard Rodent Chow than Balanced Diet (Supp. Table 1, p = 0.008, two-tailed t-test, FDR corrected). Endocannabinoids 2-AG and AEA did not differ between the diets in torpor or IBA (Supp. Tables 1 and 2).

As a follow-up, we examined the influence of season and physiological state on hypothalamic endocannabinoids and NAEs. Summer Standard Rodent Chow hypothalamic samples were compared to torpor and IBA time points. AEA, OEA and synaptamide were higher in summer than in either hibernation state (Table 2, p < 0.05, one-way ANOVA, post hoc Tukey). PEA and 2-AG were increased in torpor compared to IBA (Table 2, p < 0.05, one-way ANOVA, post hoc Tukey).

**Table 2:** Seasonal changes in hypothalamic endocannabinoids and NAEs.

Seasonal hypothalamic fatty acid amides							
Bioactive fatty acid amide	State	N	Mean (pmol/nmol std. error lipid P)		p value vs Torpor	p value vs Arousal	p value vs summer
14:0-NAE	Torpor	15	0.76	0.25	n/a	0.915	0.942
	Aroused	8	0.62	0.24	0.915	n/a	0.798
	Summer	8	0.88	0.17	0.942	0.798	n/a
16:1-NAE	Torpor	15	0.40	0.09	n/a	0.822	0.341
	Aroused	8	0.33	0.03	0.822	n/a	0.197
	Summer	8	0.58	0.11	0.341	0.197	n/a
PEA (16:0-NAE)	Torpor	15	9.99*	1.39	n/a	0.008	0.337
	Aroused	8	4.11	0.60	0.008	n/a	0.256
	Summer	8	7.41	0.90	0.337	0.256	n/a
Linoleoylethanolamide (18:2-NAE)	Torpor	15	1.23	1.10	n/a	0.775	0.994
	Aroused	8	0.28	0.14	0.775	n/a	0.770
		8	1.37	0.70		0.770	n/a
	Summer				0.994		
OEA (18:1-NAE)	Torpor	15	8.33	1.40	n/a	0.856	0.031
	Aroused	8	7.32	0.67	0.856	n/a	0.023
	Summer	8	13.40*	1.22	0.031	0.023	n/a
Stearoylethanolamide (18:0-NAE)	Torpor	15	4.93	2.29	n/a	0.594	0.901
	Aroused				0.594		
		8	2.16	0.57		n/a	0.882
		8	3.70	0.79		0.882	n/a
	Summer				0.901		
AEA (20:4NAE)	Torpor	15	0.13	0.02	n/a	0.995	0.005
	Aroused	8	0.13	0.02	0.995	n/a	0.017
	Summer	8	0.22*	0.02	0.005	0.017	n/a
20:1-NAE	Torpor	15	0.24	0.04	n/a	0.604	0.538
	Aroused	8	0.18	0.03	0.604	n/a	0.193
	Summer	8	0.30	0.05	0.538	0.193	n/a
20:0-NAE	Torpor	15	0.43	0.08	n/a	0.459	0.739
	Aroused	8	0.31	0.05	0.459	n/a	0.914
	Summer	8	0.36	0.05	0.739	0.914	n/a
Synaptamide (22:6-NAE)	Torpor	15	0.04	0.01	n/a	0.939	0.001
	Aroused	8	0.04	0.01	0.939	n/a	0.002
	Summer	8	0.09*	0.01	0.001	0.002	n/a
22:1-NAE	Torpor	15	0.12	0.01	n/a	0.104	0.425
	Aroused	8	0.08	0.02	0.104	n/a	0.740
	Summer	8	0.10	0.01	0.425	0.740	n/a
22:0-NAE	Torpor	15	0.37	0.10	n/a	0.444	0.231
	Aroused	8	0.55*	0.15	0.444	n/a	0.043
	Summer	8	0.12	0.04	0.231	0.043	n/a
24:0-NAE	Torpor	15	0.13	0.02	n/a	0.975	0.836
	Aroused	8	0.14	0.05	0.975	n/a	0.947
	Summer	8	0.15	0.04	0.836	0.947	n/a
2-AG	Torpor	15	543.74*	72.54	n/a	0.037	0.468
	Aroused	8	307.48	30.08	0.037	n/a	0.440
	Summer	8	435.75	27.65	0.468	0.440	n/a

Synaptamide, AEA and OAE decrease during the hibernation season (torpor and arousal vs. summer) (post hoc Tukey following ANOVA, p < 0.05, n = 8–15). PEA and 2-AG are both higher in torpor than arousal (post hoc Tukey following ANOVA, p < 0.05, n = 8–15). SRC summer measurements were compared to torpor and IBA. Torpor and IBA measurements combined Balanced Diet and Standard Rodent Chow.

## Discussion

Feeding increased omega 3 PUFAs prior to hibernation, with a more balanced omega 6:3 ratio, positively modulates thermogenesis in torpor when animals fast and live off fat stores. This effect could be mediated by increasing BAT mass accumulation and increasing DHA and EPA levels in plasma, WAT and BAT. The altered diet did not influence hypothalamic endocannabinoids during the hibernation season, as initially hypothesized, but did alter some saturated hypothalamic NAEs during torpor. Omega 3 PUFAs are prominent in free-ranging hibernator diets^11,39,40^, and we show are liberated during the major thermogenic and energetically costly^3^ periods of hibernation (interbout arousal). Omega 3 PUFAs therefore may represent a dietary factor which could directly support critical thermogenic function during the hibernation season.

BAT adipogenesis, evidenced by the increase in axillary BAT mass in Balanced Diet AGS, may arise prior to or during hibernation. In non-hibernators omega 3 PUFAs enhance BAT adipogenesis via cAMP activation and upregulation of microRNAs^15^. Further, recent studies have found agonists of FFAR4, such as DHA, directly increase transcriptional programming for BAT adipogenesis in non-hibernating species^15,43,44^. As BAT is a major contributor of thermogenesis in hibernation we propose the higher levels of DHA in Balanced Diet AGS drives BAT adipogenesis resulting in the larger BAT deposits that likely contribute to the overall higher AGS T_b_ in torpor. As animals were kept at 2 °C, increased T_b_ in torpor requires an increase in metabolism.

One of the most striking characteristics of Balanced Diet AGS plasma are high levels of DHA and EPA compared to Standard Rodent Chow fed AGS in torpor. We propose circulating DHA and EPA may also stimulate thermogenesis in Balanced Diet animals. Although other FFAs can also directly stimulate uncoupling protein 1 (UCP1) in BAT, no difference in total plasma FFA, PUFA or MUFA was found between the diet groups indicating thermogenic mechanisms did not depend on changes in total concentration of FFA^23^. While multiple fatty acids have signaling properties, the fivefold increase in plasma DHA in torpor stands in contrast to the lack of difference in plasma omega 6 cascade metabolites (LA, ARA, DPA ω6) or total PUFAs levels between the two diets. This suggests either lipolysis and release of DHA and EPA into circulation during hibernation in the Balanced Diet AGS or increased production of DHA and EPA through altered enzymatic activity of elongation and desaturation for the omega 3 synthesis pathway in Balanced Diet AGS. Enhanced production of omega 3 long chain PUFAs is supported in other species as DHA in marmot cardiac and liver phospholipid membranes increases over the hibernation season^40^. Further, Arnold et al. (2011) hypothesized these omega 3 PUFAs may exert some thermogenic influence on timing of interbout arousal^40^.

DHA itself is also a powerful modulator of many signaling cascades^45–47^. Previous studies in non-hibernators have shown omega 3 PUFA intake (via DHA and EPA) increases mitochondrial respiration in BAT, stimulates the beiging of white adipose tissue (WAT) to thermogenically active tissue, and increases whole body thermogenesis^13,15–17,43,48^. Proposed mechanisms of action include stimulation of omega 3 PUFA receptor FFAR4 (GPR120) or modulation of thermosensing channel TRPV1 with downstream activation of fibroblast growth factor-21 (FGF21)^13,15,44,49^. FFAR4 is a G-protein coupled receptor highly expressed in fat tissues^49^. When bound by omega 3 PUFAs, FFAR4 activates a Ca^2+^ signaling cascade that sensitizes insulin signaling and inhibits inflammation^49–51^. PUFAs, such as LA and DHA, are also known to bind and activate other free fatty acid receptors such as FFA1^52^. Former studies show treatment with mercaptoacetate, a deregulator of beta-oxidation and a known antagonist of FFA1 (GPR40)^53^, altered thermoregulation in hibernating ground squirrels^54^. Therefore, multiple fatty acid receptors could be targets of future investigations on signaling mechanisms for thermogenesis in hibernation.

A preference for the omega 3 synthesis pathway in hibernation, as well as active metabolism of omega 3 PUFAs, is indicated by seasonally high plasma DHA in both feed groups in torpor compared to fall levels. In contrast, ω6 DPA decreases and ARA remains unchanged in torpor compared to fall in both diets. As the major source of the omega 3 PUFA in Balanced Diet is ALA, the decrease in BAT ALA in IBA compared to torpor potentially indicates consumption of the omega 3 PUFA. Non-hibernator BAT cells, but not WAT, have been shown to produce DHA^55^.

A preference for PUFA metabolism also appears during interbout arousal from torpor. Our data shows plasma PUFA metabolites increase up to eightfold during natural early interbout arousal when the majority of thermogenesis is thought to be produced from BAT^42,56^. While lipolysis is part of the endogenous arousal process and many free fatty acids are released in early interbout arousal, the increase in DHA, EPA, and ARA are significantly higher than their parent compounds or MUFAs or SFAs and remain elevated into the second phase of early interbout arousal. Curiously, bats were found to oxidize PUFAs significantly more than SFAs in hibernation, potentially supporting a more dynamic use of PUFAs in hibernation than previously considered^57^. The sustained increase in long chain PUFAs, such as DHA, could further be explained by the fact ALA β-oxidation kinetics are known to be faster than other fatty acids in mitochondria^58^.

A question our study raises is if free-ranging hibernators actively use omega 3 PUFAs as thermogenic mediators during the winter. Arnold et al. (2011) documented systemic omega 3 PUFA loss from tissue stores in free-ranging marmots over a hibernation season barring an accumulation of DHA in cardiac and liver phospholipids^40^. Theoretically, DHA and EPA production during natural interbout arousal may constitute part of a signaling mechanism to enhance thermogenesis or BAT mass. Given free-ranging hibernators consume omega 3 PUFAs, omega 3 stimulated BAT accumulation could additionally be beneficial for cold challenged, free-ranging animals. In the wild, AGS encounter ambient temperatures that dip as low as − 19 °C^2^. To defend a T_b_ greater than − 3 °C, they increase metabolism and thermogenesis^59,60^. Cold challenge, known to increase metabolism, also activates FFAR4 in BAT, indicating a linked mechanism to omega 3 thermogenesis^15,43,44,49,61^. Greater deposition of BAT could be a defense mechanism against this natural environmental challenge^42,62^.

While we propose omega 3 PUFAs play a role in BAT accumulation and thermogenesis in hibernation, altered PUFA phospholipid membrane incorporation may also impact metabolism and thermoregulation^63^ Arnold et al. hypothesized phospholipid composition is specifically retained at a high omega 6:3 ratio for cardiac function at low temperature and to enhance sacro/endoplasmic reticulum Ca^2+^-ATPase (SERCA) function^63–65^. As we show in BAT, WAT and plasma, Balanced Diet lowered the omega 6:3 PUFA ratio by over half, suggesting a physiologically significant change we expect to be present in phospholipid membrane incorporation as well. Further work would need to discriminate the possible influence of Balanced Diet on phospholipid composition, free fatty acids in plasma and triglyceride composition.

Interestingly, our findings differed from some previous omega 3 hibernation studies^7,11,12^. One study found feeding higher ratios of omega 3 PUFAs than we employed caused marmots to maintain euthermic T_b_ and not hibernate throughout the entire hibernation season^11^. This suggests that there may be a concentration dependent influence of omega 3 PUFAs on hibernation. Further, two hibernation studies found no influence of omega 3 PUFAs on thermogenesis^7,12^, suggesting there may be species specific reactions to omega 3 PUFA feeding as well. Further, while increased T_b_ is often associated with shorter torpor bouts^10^, our increase in T_b_ did not translate to shorter torpor bout lengths. While this could suggest there may be more efficiency in energy expenditure in Balanced Diet AGS, further studies are required. An alternative explanation could be that the increase in T_b_ was not extensive enough to influence torpor bout length duration. Secondly, because tissues were collected mid-December, it is possible that an influence on torpor bout length would appear later in the hibernation season. Our results correspond with Frank et al. who found feeding higher levels of omega 3 PUFAs did not influence torpor bout length^7^.

Our results did not support our hypothesis that Balanced Diet would influence hypothalamic endocannabinoid and bioactive NAE levels, but data does show seasonal trends in endocannabinoids with some differences in saturated NAEs between diets. PEA (16:0 NAE), a bioactive fatty acid amide that is close in structure to AEA, and 2-AG, a full agonist of the CB1 receptor, increased in torpor in AGS compared to IBA, but did not differ from summer, potentially representing a circannual rhythm between the hibernation phases. As 2-AG is thought to modulate metabolism and induce hypothermia^32^, an intriguing question is whether increased 2-AG in torpor has any physiologic effect, such as reduction in body temperature. Additionally, the relationship between torpid and IBA 2-AG levels is somewhat similar to certain fasting experiments which saw increased 2-AG during severe fasting and reduction of 2-AG with feeding^31^. PEA on the other hand is not known to directly activate CB_1_ or CB_2_ receptors, but has been shown to desensitize TRPV1, activate PPARα and PPARγ as well as have strong anti-inflammatory and analgesic activity^28^. The seasonal increase in PEA may represent a protective mechanism given its anti-inflammatory and analgesic properties. While a recent study documented no significant change in hypothalamic endocannabinoids in marmots between summer and hibernation, they did see significant decreases in endocannabinoids in other tissues^35^. In conjunction with our findings, a lack of depression in multiple hypothalamic endocannabinoids and NAEs between both our studies (excluding the decreases in AEA, OAE and synaptamide) is intriguing in that most metabolic functions are profoundly downregulated in torpor. Given the unique position the endocannabinoid system holds in metabolism, satiation, thermogenesis, synaptic plasticity and 2-AG and PEA’s oscillation in hibernation, the physiologic role of endocannabinoids in hibernation beyond omega 3 dietary influence requires further study.

In conclusion, dietary omega 3 PUFAs, such as DHA and EPA, may play a beneficial role in increasing thermogenic capacity in hibernators. Increased BAT accumulation driven by dietary omega 3 PUFAs may represent a natural mechanism for AGS to increase BAT stores prior to and during hibernation.

## Methods

### Animal trapping

Juvenile AGS were lived-trapped during July on the northern side of the Brooks Range in Alaska, forty miles south of Toolik Field Station (64° 86′ N, 147° 84′ W). AGS were transported to the University of Alaska Fairbanks under IACUC approved protocols and Alaska Fish and Game permits.

### Husbandry

Captive AGS were housed individually in 30 cm × 48.3 cm × 30 cm stainless steel wire mesh hanging cages with cotton nests. Cages were hung over ammonia absorbing corn cob litter. AGS were housed at ambient temperature (T_a_, 16–18 °C) and 16L:8D hour light/dark cycle until August 15th, when they were moved to cold chambers with T_a_ of 2 °C at a 4L:20D hour light/dark cycle. In mid-July, animals were offered 47 g daily of either Standard Rodent Chow (#5663, Mazuri, PMI Nutrition International, Richmond, IN) or Balanced Diet (9GU5, formulated with Lab Diet, St. Louis, MO) and were provided water *ad litbitum* during the euthermic period (Table 1b). Once animals began hibernating, food was withdrawn. At this time animals were placed in polycarbonate cages (21.6 cm × 43.2 cm × 21.6 cm) with shavings, cotton bedding and gel hydration packets. The majority of AGS entered hibernation in September. Feed groups were evenly staggered on racks to ensure micro-climates in the cold chamber did not confound effects of diet on core body temperature (T_b_). Care was taken to minimize all disturbances in the hibernation chamber during the hibernation season and access was restricted to research and veterinary staff per University of Alaska Fairbanks Institutional Animal Care and use Committee (IACUC) approved protocols.

### Diet

Both diets were stored at − 4 °C. The contents of the Balanced diet (9GU5, formulated by Lab Diet, St. Louis, MO) provided by the manufacturer were 23.4% protein, 6.5% fat, 6% ash, 3.3% fiber. The contents of the Standard Rodent Chow (#5663, Mazuri, PMI Nutrition International, Richmond, IN) provided by the manufacturer were 23% protein, 6.5% fat, 4.5% fiber, 6.5% ash. Basic fatty acid composition, determined by GC as described below, of both diets is provided (Table 1b).

### Animals

All procedures were performed and approved in accordance with University of Alaska Fairbanks Institutional Animal Care and use Committee and the Guide for Care and Use of Laboratory Animals (Eighth edition). Each dietary group had 6 juvenile males and 6 juvenile females. One animal died in the fall for reasons unrelated to diet. All animals remaining hibernated through the winter.

### Surgery

During the pre-hibernation feed period, waxed and sterilized I-button temperature loggers (DS1922L-F5 temperature accuracy from − 10 to + 65 °C, resolution ± 0.5 °C, Maxim Integrated, San Jose, CA) were implanted in the abdominal cavity using sterile technique as previously described^66^. Loggers were recovered after tissue collection in winter and temperature data were downloaded. I-button sampling rate was every 30 min throughout the hibernation season.

### Blood and tissue collection

During the hibernation season, the “shavings added” method was employed to track torpor bout length^66^. On the first day of torpor when respirations were below 5 breaths per minute and the animal was inactive and showed a curled posture, shavings were placed onto the animals’ back. When animals arouse during IBA, shavings are disturbed. Using this method to identify when animals were hibernating, we monitored the length of torpor bouts. Animals were checked daily through the entire hibernation season. Torpor bout length and hibernation parameters were verified by I-button temperature data after euthanasia. Tissues were collected mid-December during three distinct physiological states of the hibernation torpor bout: Early torpor (defined as 10–25% of torpor bout length based on the average of the previous two torpor bouts, n = 7), late torpor (defined as 89–100% of torpor bout length based on the average of the previous two torpor bouts, n = 8) and IBA (core body temperature, (T_b_) above 33 °C, n = 8). All torpid animals were euthanized between 9 and 11 am. Interbout arousal was induced by handling at 9 am, IBA tissues were collected at 1 pm.

To compare mass of BAT between animals we needed to collect all BAT tissue within the same seasonal time frame (mid-December). For this reason, we chose to induce arousal by brief, gentle, handling. Arousal induced by handling induces thermogenesis faster than natural arousal, but total thermogenesis is expected to be similar between handling-induced and natural arousal. For this reason, we do not think that handling animals to induce arousal confounds our interpretation of results.

Prior to tissue collection, euthermic animals were anesthetized with isoflurane (5% mixed with medical grade oxygen and delivered at 1.5 L/min) to achieve a surgical plane of anesthesia. Blood was sampled by cardiac puncture within 3 min after removing AGS from the home cage and a rectal thermometer was placed in the AGS. Animals (n = 23) were immediately decapitated after blood collection and tissues were dissected, wrapped in foil and placed immediately on dry ice. Brown adipose tissue (BAT) axillary deposits were easily dissected from surrounding tissue and weighed. Visceral white adipose tissue (WAT) was sampled from the abdominal cavity without difficulty. Fall plasma and visceral WAT were opportunistically sampled in August during surgery (approximately 3 weeks into the start of the feed trial), but BAT was not sampled in fall to reduce stress on the animals. Summer hypothalamus measurements for endocannabinoids came from a separate group of AGS fed Standard Rodent Chow Diet.

### Hibernation physiology

T_b_ was monitored to assess the impact of diet on hibernation. AGS T_b_ in torpor was reported as the average T_b_ during torpor, defined as a T_b_ less than 4 °C after animals had their first two torpor bouts. AGS T_b_ in interbout arousal was reported as the average T_b_ after animals had their first two torpor bouts when animal’s T_b_ was over 34 °C. AGS torpor bout length was reported as hours from entrance to torpor to exit from torpor when animal’s T_b_ was below 5 °C. Torpor bouts last approximately 14–21 days after the first initial torpor bouts. Torpor is interrupted by interbout arousal periods (IBA) that last approximately 24 h (Fig. 2d).

### Lipid fatty acid analysis

100 µl of plasma was thawed and decanted into a screw top test tube. 100 μg of free nonadecanoic acid (19:0) was added as an internal fatty acid standard (odd chain fatty acids are found in low abundance in non-rumen animals). Plasma lipids were then converted to fatty acid methyl esters with the addition of sulfuric acid in methanol and heated for 60 min at 100 °C^67^. Fatty acid methyl esters were extracted from sample tubes with the addition of water and distilled petroleum ether (mixed hexanes). The resulting non-polar ether phase was decanted and dried down under a gentle stream of nitrogen at ambient temperature.

Dried samples were immediately reconstituted with 100 µl of petroleum ether and decanted to a sample vial. Fatty acid methyl esters were then analyzed with a Shimadzu gas chromatograph (GC) model 2010. Samples were injected in split mode into a Restek (Bellefonte, PA) FAMEWAX 30 m column. The GC was programmed from 160 to 220 °C and detection conducted with flame ionization detection (FID). White adipose tissues were processed as described above with additional vortexing. For chow analysis, chow was homogenized with a standard coffee grinder into a fine particulate. The fatty acid and fat content of chow were determined as detailed above. Chromatograms and data were reviewed and calculated with Shimadzu Class VP software. Data are expressed as µg fatty acid per ml of plasma or mg of tissue (wet weight).

### NAE and endocannabinoid hypothalamic analysis

Hypothalamus (5–10 mg excision) samples were subjected to Bligh and Dyer lipid extraction^67^ using Barocycler 2320EXT apparatus (Pressure Biosciences, Inc., Medford, MA), Teflon tubes with 0.15 ml with microcaps, and cycling pressure mode. Tubes were filled with 0.15 ml methanol/chloroform/0.9%KCl (2:1:0.5, v/v) and subjected to 30 pressure cycles of extraction (20 s at 35,000 psi then 20 s at 0 psi) at 4 °C. After pressure-assisted extraction, extracts were transferred into 8-ml glass screw-capped tubes with methanol, chloroform, and 0.9%KCl to produce a total of 2 mL methanol, 1 mL chloroform, and 0.5 mL 0.9%KCl. At this step, 20 pmol d5-2AG, 20 pmol d4-anandamide (both from Cayman Chemicals, Ann Arbor, MI) and 20 ng d4-NAE internal standard mix (made by transamination of a mixture of triglycerides and d4-ethanolamine (130 °C × 4 h) with subsequent d4-NAE precipitation from acetone and calibration by gas chromatography) were added. Extraction was finalized by the addition of 1 mL chloroform, 1.3 mL 0.9%KCl, vortexing and centrifugation (2000×*g* × 15 min). Chloroform phase was collected, evaporated by the stream of nitrogen, and re-dissolved in exactly 1 mL volume of methanol/chloroform (9:1, v/v). Extract aliquots (0.01 mL) were taken and total lipid phosphorus content was measured as described^68^. At the end, extracts were evaporated again, re-dissolved in 0.15 mL methanol and transferred into injection vials for NAE-2AG analysis by liquid chromatography electrospray ionization tandem mass spectrometry (LC–MS/MS).

LC–MS/MS of NAE and 2-AG was performed using AB Sciex 6500QTRAP mass spectrometer coupled with Shimadzu Nexera X2 UHPLC system. NAE and 2-AG were resolved on Ascentis Express RP-Amide 2.7 μm 2.1 × 50 mm column using gradient elution from methanol/water/formic acid (65:35:0.5, 5 mM ammonium formate) to methanol/chloroform/water/formic acid (90:10:0.5:0.5, 5 mM ammonium formate). NAEs were detected in a positive ions mode as a transition from corresponding molecular ions to m/z 62 (natural NAEs) or m/z 66 (d4-NAE); 2-AG was detected in positive ions mode as a transition from m/z 379 to m/z 287, and d5-2AG was detected as a transition from m/z 384 to m/z 287. Each analyte was detected based on principles previously described^69,70^ and quantified using isotope dilution approach and normalized per total sample lipid phosphorus content.

### Undisturbed sampling of plasma during natural interbout arousal

Another set of AGS were instrumented with chronic femoral arterial and venous cannulas (3 Fr cannula, Instech Laboratories Inc, Plymouth Meeting, PA, USA) and either TA-F40 or CTA-F40 core body temperature (T_b_) loggers implanted in the abdominal cavity (Data Sciences International, St. Paul, MN, USA, calibrated to 0, 35 and 39 °C, resolution ± 0.01 °C) in July and August as described previously^41^. Tb was recorded throughout the hibernation season at intervals of every 10 min. Cannulas were maintained with a heparin/glycerol locking solution (1:1). These animals were solely used for undisturbed sampling of plasma during natural IBA from torpor and were fed Standard Rodent Chow (n = 9). Blood was sampled during a torpor bout and through a natural IBA without disturbing AGS (female n = 5, male n = 4). Blood sampled at four time points of natural interbout arousal: Early arousal (T_b_ 2.5 °C), early arousal (T_b_ 4 °C), mid arousal (T_b_ 16 °C) and full arousal (T_b_ 35 °C). To delineate early lipolysis fueling interbout arousal we sampled two early arousal time points.

### Ultrahigh-performance liquid chromatograph mass spectrometry (UHPLC-MS) analysis

Plasma samples (20 µL) were extracted using ice cold methanol:acetonitrile:water (5:3:2) at 1:25 dilution as previously described^71^. Extracts were analyzed on a Thermo Vanquish ultra-high performance liquid chromatograph (UHPLC) coupled online to a Thermo Q Exactive mass spectrometer using a 5 min C18 gradient method in positive and negative modes as described previously^72,73^. Peak picking and metabolite assignment were performed using Maven (Princeton University) against the KEGG database^74–76^, confirmed against chemical formula determination from isotopic patterns and accurate mass, and validated against experimental retention times for > 650 standard compounds (Sigma Aldrich; MLSMS, IROATech, Bolton, MA, USA)^77^.

### Statistical analysis

Results were analyzed using SPSS statistical package (IBM, v. 25) and MetaboAnalyst 4.0. Data are shown as mean ± standard error of the means (SEM). Data were checked for normal distribution. Two-tailed t-test results that violated Levene’s test for equality of variances were reported with equal variances not assumed. T-tests were reported with false discovery rate (FDR) correction (p < 0.05) based on the Benjamini–Hochberg procedure. ANOVAs were reported with post hoc Tukey correction. Late torpor and early torpor samples were combined because fatty acid and endocannabinoid levels did not significantly differ between early and late torpor (t-test with FDR correction). Pathway enrichment analysis and PLS-DA were performed using MetaboAnalysist 4.0^78,79^.

## Supplementary Information

Supplementary Information.

## Data Availability

The datasets generated during and/or analyzed during the current study are available from the corresponding author on reasonable request.

## Abbreviations

BD: Balanced diet
SRC: Standard Rodent Chow
AGS: Arctic Ground Squirrel
BAT: Brown Adipose Tissue
WAT: White Adipose Tissue
T_b_: Core body temperature
IBA: Interbout arousal
2-AG: Arachidonoylglycerol
AEA: Anandamide
NAEs: *N*-Acylethanolamides
PUFA: Polyunsaturated fatty acid
FFA: Free fatty acid
SFA: Saturated fatty acid
DHA: Docosahexaenoic acid
EPA: Eicosapentaenoic acid
ARA: Arachidonic acid
ALA: Alpha linolenic acid
LA: Linoleic acid
