# Supplementary material for: Omega 3 fatty acids stimulate thermogenesis during torpor in the Arctic Ground Squirrel
Source: Sci Rep. 2021 Jan 14;11:1340. doi: 10.1038/s41598-020-78763-8 (PMC7809411; doi:10.1038/s41598-020-78763-8)
Supplement: Supplementary file 1 — Supplementary Information. [file 41598_2020_78763_MOESM1_ESM.pdf]

Supplemental Figures and Tables for Manuscript

Title: Omega 3 Fatty Acids Stimulate Thermogenesis During Torpor in the Arctic Ground Squirrel

Authors: Rice, SA[1,2]; Mikes, M[2], Bibus, D[3]; Berdyshev, E[4]; Reisz, JA[5]; Gehrke, S[5]; Bronova, I[4]; D'Alessandro, A[5]; Drew, KL[1,2*]

[1]Department of Chemistry and Biochemistry, [2]Institute of Arctic Biology, Center of Transformative Research in Metabolism, University of Alaska Fairbanks, Fairbanks, AK, United States [3]Lipid Technologies, LLC, Austin, MN, United States [4]National Jewish Health, Denver, CO, United States [5]Department of Biochemistry and Molecular Genetics, University of Colorado Anschutz Medical Campus, Aurora, CO, United States

Corresponding author: kdrew@alaska.edu

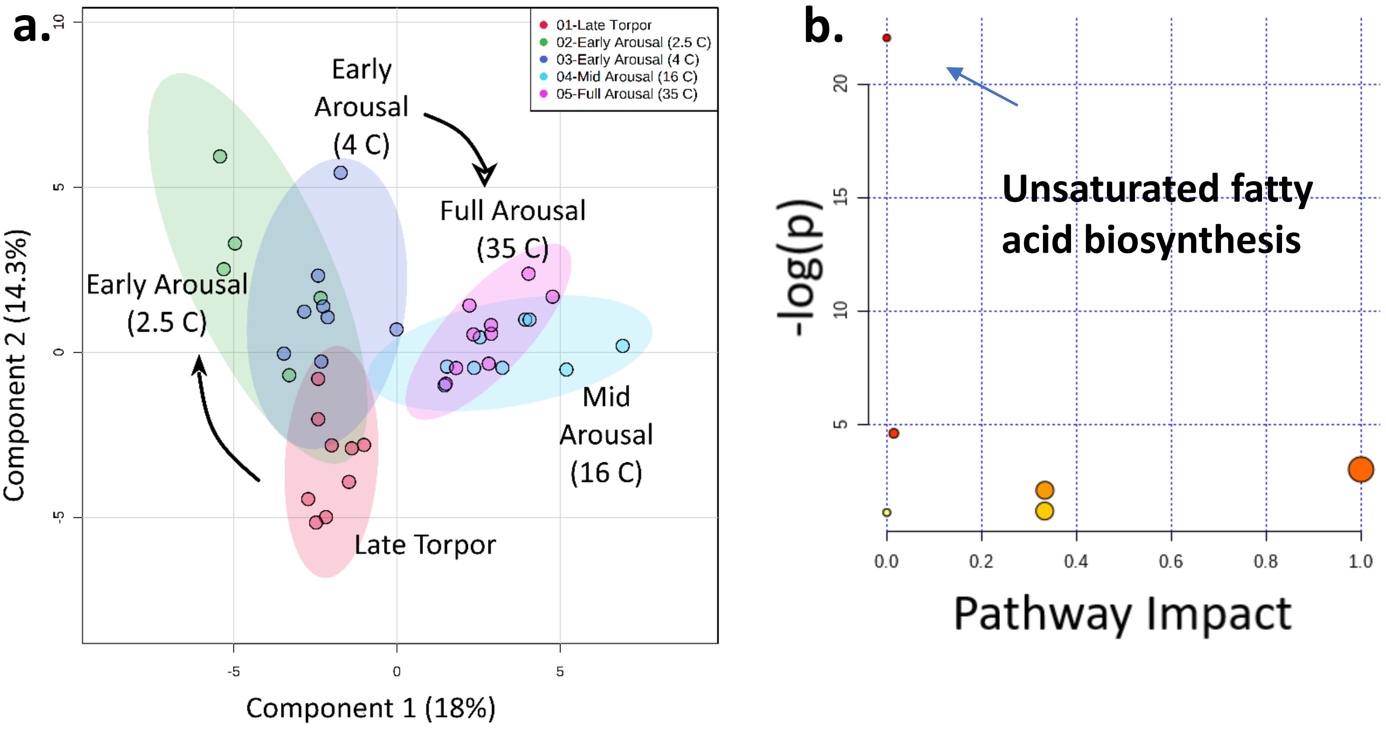

Supplemental Figure 1. Plasma metabolomic analysis during natural arousal from torpor shows spike of PUFA metabolites during early arousal a. Results indicate metabolite clustering based on phase of arousal from torpor using partial least-squares-discriminant analysis (PLS-DA). PLS-DA is a multivariant classification method that distinguishes differences between groups (specific phases of arousal). The dots are weighted averages of variables which describe the differences between groups. Results show phases of arousal cluster into unique, distinct lipid groups. **b.** Biosynthesis of unsaturated fatty acids dominate pathway analysis of plasma lipid profiling of natural arousal (utilizing p values <0.05 from FDR corrected t-tests between early arousal $4^{o}C$ and full arousal $35^{o}C$). Pathway enrichment analysis, utilizing lipids which significantly change over arousal, indicates biosynthesis of PUFAs the most significant lipid metabolic change during arousal from torpor ($p<0.0001$, $n=9$). The MetaboAnalyst visual representation of these significant changes come from combining significant p values from pathway enrichment analysis and pathway topology analysis. **c.** Measurements are from individual animals sampled over a single, undisturbed natural arousal fed Standard Rodent Chow. Blood was sampled at specific core body temperatures during arousal ranging from early arousal ($2.5^{o}C$) to full arousal ($35^{o}C$).

Torpid Hypothalamic Fatty Acid Amides					
**Fatty Acid Amides**	Diet	N	Mean (pmol/nmol lipid P)	Std. Error Mean	P value (FDR corrected)
**14:0-NAE**	Balanced Diet	8	**1.42***	0.31	0.008*
	Standard Rodent Chow	7	0.01	0.01	
**16:1-NAE**	Balanced Diet	8	0.52	0.15	0.574
	Standard Rodent Chow	7	0.28	0.03	
**PEA (16:0-NAE)**	Balanced Diet	8	12.82	2.05	0.104
	Standard Rodent Chow	7	6.75	0.86	
**linoleoylethanolamide (18:2-NAE)**	Balanced Diet	8	2.22	2.05	0.602
	Standard Rodent Chow	7	0.09	0.03	
**OEA (18:1-NAE)**	Balanced Diet	8	9.78	2.51	0.576
	Standard Rodent Chow	7	6.67	0.77	
**Stearoylethanolamide (18:0-NAE)**	Balanced Diet	8	7.68	4.17	0.576
	Standard Rodent Chow	7	1.78	0.29	
**AEA (20:4NAE)**	Balanced Diet	8	0.12	0.01	0.764
	Standard Rodent Chow	7	0.13	0.04	
**20:1-NAE**	Balanced Diet	8	0.26	0.07	0.749
	Standard Rodent Chow	7	0.22	0.03	
**20:0-NAE**	Balanced Diet	8	0.50	0.13	0.602
	Standard Rodent Chow	7	0.36	0.06	
**Synaptamide (22:6-NAE)**	Balanced Diet	8	0.04	0.01	0.963
	Standard Rodent Chow	7	0.05	0.01	
**22:1-NAE**	Balanced Diet	8	0.12	0.02	0.749
	Standard Rodent Chow	7	0.13	0.02	
**22:0-NAE**	Balanced Diet	8	**0.11***	0.04	0.008*
	Standard Rodent Chow	7	0.66	0.13	
**24:0-NAE**	Balanced Diet	8	0.10	0.02	0.576
	Standard Rodent Chow	7	0.15	0.04	
**2-AG**	Balanced Diet	8	509.78	30.30	0.749
	Standard Rodent Chow	7	582.55	156.90	

Supplemental Table 1. Balanced Diet increases torpid hypothalamic 22:0 and 14:0 NAE the fatty acid amides, but does not influence bioactive NAEs and endocannabinoids compared to Standard Rodent Chow (two-tailed t-test, FDR corrected, n=7-8).

Interbout Arousal Hypothalamic Fatty Acid Amides					
**Fatty Acid Amides**	Diet	N	Mean (pmol/nmol lipid P)	Std. Error Mean	P value (FDR corrected)
**14:0-NAE**	Balanced Diet	4	1.15	0.30	0.185
	Standard Rodent Chow	4	0.09	0.07	
**16:1-NAE**	Balanced Diet	4	0.35	0.04	0.781
	Standard Rodent Chow	4	0.31	0.04	
**PEA (16:0-NAE)**	Balanced Diet	4	5.21	0.80	0.384
	Standard Rodent Chow	4	3.01	0.49	
**linoleoylethanolamide (18:2-NAE)**	Balanced Diet	4	0.16	0.11	0.781
	Standard Rodent Chow	4	0.40	0.26	
**OEA (18:1-NAE)**	Balanced Diet	4	7.70	1.02	0.781
	Standard Rodent Chow	4	6.94	0.98	
**Stearoylethanolamide (18:0-NAE)**	Balanced Diet	4	2.39	0.73	0.831
	Standard Rodent Chow	4	1.93	0.97	
**AEA (20:4NAE)**	Balanced Diet	4	0.12	0.03	0.781
	Standard Rodent Chow	4	0.14	0.03	
**20:1-NAE**	Balanced Diet	4	0.23	0.05	0.384
	Standard Rodent Chow	4	0.14	0.03	
**20:0-NAE**	Balanced Diet	4	0.40	0.08	0.384
	Standard Rodent Chow	4	0.22	0.03	
**Synaptamide (22:6-NAE)**	Balanced Diet	4	0.03	0.02	0.674
	Standard Rodent Chow	4	0.05	0.01	
**22:1-NAE**	Balanced Diet	4	0.10	0.03	0.384
	Standard Rodent Chow	4	0.05	0.01	
**22:0-NAE**	Balanced Diet	4	0.60	0.22	0.854
	Standard Rodent Chow	4	0.51	0.23	
**24:0-NAE**	Balanced Diet	4	0.17	0.09	0.781
	Standard Rodent Chow	4	0.10	0.04	
**2-AG**	Balanced Diet	4	304.86	59.90	0.939
	Standard Rodent Chow	4	310.09	25.09	

Supplemental Table 2. Diet does not influence hypothalamic fatty acid amides or endocannabinoids during interbout arousal (two-tailed t-test, n=7-8, FDR corrected).

Plasma Early Torpor Fatty Acids

Fatty Acid	Diet	N	Mean (µg/ml)	Std. Error Mean
Linoleic Acid (18:2ω6)	Balanced Diet	4	4208.85	892.98
	Standard Rodent Chow	3	3164.77	109.92
Alpha Linolenic Acid (18:3ω3)	Balanced Diet	4	400.06	105.07
	Standard Rodent Chow	3	148.92	25.81
Arachidonic Acid (20:4ω6)	Balanced Diet	4	485.06	20.95
	Standard Rodent Chow	3	383.96	49.36
EPA (20:5ω3)	Balanced Diet	4	114.37	14.24
	Standard Rodent Chow	3	8.00	0.90
DHA (22:6ω3)	Balanced Diet	4	581.25	73.45
	Standard Rodent Chow	3	80.53	17.54
DPA ω6 (22:5 ω6)	Balanced Diet	4	12.65	1.83
	Standard Rodent Chow	3	9.88	2.37

Plasma Late Torpor Fatty Acids

Fatty Acid	Diet	N	Mean (µg/ml)	Std. Error Mean
Linoleic Acid (18:2ω6)	Balanced Diet	4	3845.02	255.45
	Standard Rodent Chow	4	3894.54	57.42
Alpha Linolenic Acid (18:3ω3)	Balanced Diet	4	210.20	39.19
	Standard Rodent Chow	4	147.38	20.13
Arachidonic Acid (20:4ω6)	Balanced Diet	4	497.30	57.59
	Standard Rodent Chow	4	488.27	31.33
EPA (20:5ω3)	Balanced Diet	4	113.13	23.23
	Standard Rodent Chow	4	9.17	0.79
DHA (22:6ω3)	Balanced Diet	4	493.59	65.00
	Standard Rodent Chow	4	112.40	12.01
DPA ω6 (22:5 ω6)	Balanced Diet	4	10.48	1.99
	Standard Rodent Chow	4	15.77	1.05

Supplemental Table 3. Data show exact free fatty acid values in plasma in late torpor and early torpor AGS. Values were not significantly different between late torpor and early torpor (FDR corrected t-test).

WAT Early Torpor Fatty Acids

Fatty Acid	Diet	N	Mean (μg/mg)	Std. Error Mean
Linoleic Acid (18:2ω6)	Balanced Diet	4	119,646	2075
	Standard Rodent Chow	3	213451	11491
Alpha Linolenic Acid (18:3ω3)	Balanced Diet	4	66347	2249
	Standard Rodent Chow	3	40464	632
Arachidonic Acid (20:4ω6)	Balanced Diet	4	1128	61
	Standard Rodent Chow	3	1201	48
EPA (20:5ω3)	Balanced Diet	4	1750	110
	Standard Rodent Chow	3	282	27
DHA (22:6ω3)	Balanced Diet	4	3363	400
	Standard Rodent Chow	3	322	54
DPA ω6 (22:5 ω6)	Balanced Diet	4	151	22
	Standard Rodent Chow	3	70	13

WAT Late Torpor Fatty Acids

Fatty Acid	Diet	N	Mean (μg/mg)	Std. Error Mean
Linoleic Acid (18:2ω6)	Balanced Diet	4	121248	2560
	Standard Rodent Chow	4	212449	7844
Alpha Linolenic Acid (18:3ω3)	Balanced Diet	4	65622	3098
	Standard Rodent Chow	4	41530	1139
Arachidonic Acid (20:4ω6)	Balanced Diet	4	1115	59
	Standard Rodent Chow	4	1571	95
EPA (20:5ω3)	Balanced Diet	4	1747	102
	Standard Rodent Chow	4	369	26
DHA (22:6ω3)	Balanced Diet	4	3255	251
	Standard Rodent Chow	4	426	132
DPA ω6 (22:5 ω6)	Balanced Diet	4	178	12
	Standard Rodent Chow	4	73	4

Supplemental Table 4. Data show exact free fatty acid values in WAT tissue in late torpor and early torpor AGS. Values were not significantly different between late torpor and early torpor (FDR corrected t-test).

BAT Early Torpor Fatty Acids

Fatty Acid	Diet	N	Mean (µg/mg)	Std. Error Mean
Linoleic Acid (18:2ω6)	Balanced Diet	4	188037	9249
	Standard Rodent Chow	3	275240	16270
Alpha Linolenic Acid (18:3ω3)	Balanced Diet	4	44919	2413
	Standard Rodent Chow	3	29449	2455
Arachidonic Acid (20:4ω6)	Balanced Diet	4	11069	2812
	Standard Rodent Chow	3	10741	2660
EPA (20:5ω3)	Balanced Diet	4	1963	411
	Standard Rodent Chow	3	378	51
DHA (22:6ω3)	Balanced Diet	4	10204	2084
	Standard Rodent Chow	3	2424	761
DPA ω6 (22:5 ω6)	Balanced Diet	4	329	53
	Standard Rodent Chow	3	411	94

BAT Late Torpor Fatty Acids

Fatty Acid	Diet	N	Mean (µg/mg)	Std. Error Mean
Linoleic Acid (18:2ω6)	Balanced Diet	4	179801	2897
	Standard Rodent Chow	4	278031	9526
Alpha Linolenic Acid (18:3ω3)	Balanced Diet	4	45503	1615
	Standard Rodent Chow	4	28030	984
Arachidonic Acid (20:4ω6)	Balanced Diet	4	8605	1245
	Standard Rodent Chow	4	12596	1538
EPA (20:5ω3)	Balanced Diet	4	2037	491
	Standard Rodent Chow	4	492	20
DHA (22:6ω3)	Balanced Diet	4	8434	1181
	Standard Rodent Chow	4	2845	235
DPA ω6 (22:5 ω6)	Balanced Diet	4	434	3
	Standard Rodent Chow	4	557	73

Supplemental Table 5 Data show exact free fatty acid values in BAT tissue in late torpor and early torpor AGS. Values were not significantly different between late torpor and early torpor (FDR corrected t-test).

Tissue	State	Comparison	Metabolite	p value (FDR Corrected)
BAT	Torpor	BD vs. SRC	w6/w3	1.02E-07
BAT	Torpor	BD vs. SRC	Linoleic Acid (182w6)	1.71E-06
BAT	Torpor	BD vs. SRC	Alpha Linolenic Acid (183w3)	3.20E-06
BAT	Torpor	BD vs. SRC	DHA (226w3)	0.000687
BAT	Torpor	BD vs. SRC	EPA (205w3)	0.001093
BAT	Torpor	BD vs. SRC	ArachidonicAcid (204w6)	0.42154
BAT	Torpor	BD vs. SRC	DPA w6(225w6)	0.20025
BAT	Arousal	BD vs. SRC	w6/w3	0.000805
BAT	Arousal	BD vs. SRC	Linoleic Acid (182w6)	0.001998
BAT	Arousal	BD vs. SRC	Alpha Linolenic Acid (183w3)	0.002854
BAT	Arousal	BD vs. SRC	Arachidonic Acid (204w6)	0.43768
BAT	Arousal	BD vs. SRC	DHA (226w3)	0.025898
BAT	Arousal	BD vs. SRC	DPA w6(225w6)	0.13869
BAT	Arousal	BD vs. SRC	EPA (205w3)	0.002854
WAT	Torpor	BD vs. SRC	w6w3	9.24E-13
WAT	Torpor	BD vs. SRC	EPA (205w3)	2.67E-09
WAT	Torpor	BD vs. SRC	Linoleic Acid (182w6)	6.28E-09
WAT	Torpor	BD vs. SRC	Alpha Linolenic Acid (183w3)	7.63E-08
WAT	Torpor	BD vs. SRC	DHA (226w3)	1.10E-07
WAT	Torpor	BD vs. SRC	DPA w6(225w6)	7.51E-05
WAT	Torpor	BD vs. SRC	Arachidonic Acid (204w6)	0.024533
WAT	Arousal	BD vs. SRC	DHA (226w3)	4.74E-07
WAT	Arousal	BD vs. SRC	EPA (205w3)	0.000674
WAT	Arousal	BD vs. SRC	Alpha Linolenic Acid (183w3)	0.001988
WAT	Arousal	BD vs. SRC	Linoleic Acid (182w6)	0.003411
WAT	Arousal	BD vs. SRC	Arachidonic Acid (204w6)	0.24198
WAT	Arousal	BD vs. SRC	w6w3	0.001456
WAT	Arousal	BD vs. SRC	Arachidonic Acid (204w6)	0.24198
Plasma	Torpor	BD vs. SRC	w6w3	1.00E-07
Plasma	Torpor	BD vs. SRC	DHA (226w3)	1.98E-05
Plasma	Torpor	BD vs. SRC	EPA (205w3)	2.49E-05
Plasma	Torpor	BD vs. SRC	Alpha Linolenic Acid (183w3)	0.11531
Plasma	Torpor	BD vs. SRC	ArachidonicAcid (204w6)	0.381
Plasma	Torpor	BD vs. SRC	Linoleic Acid (182w6)	0.42336
Plasma	Torpor	BD vs. SRC	DPA w6(225w6)	0.44594
Plasma	Arousal	BD vs. SRC	w6w3	0.00221
Plasma	Arousal	BD vs. SRC	DHA (226w3)	0.010501
Plasma	Arousal	BD vs. SRC	EPA (205w3)	0.011532
Plasma	Arousal	BD vs. SRC	Alpha Linolenic Acid (183w3)	0.17324
Plasma	Arousal	BD vs. SRC	DPA w6(225w6)	0.27087
Plasma	Arousal	BD vs. SRC	ArachidonicAcid (204w6)	0.39389
Plasma	Arousal	BD vs. SRC	Linoleic Acid (182w6)	0.40118

Supplemental Table 6. P values for two-tailed t-tests of fatty acids (FDR corrected) comparing Balanced Diet (BD) to Standard Rodent Chow (SRC) for Fig. 1, Fig. 3 and Fig. 4.

Fatty Acid	Balanced Diet Fatty Acid Percent	Standard Rodent Chow Fatty Acid Percent
10:0	0.00	0.00
12:0	0.05	0.03
14:0	0.75	0.11
14:1	0.02	0.00
15:0	0.01	0.01
15:1	0.00	0.00
16:0	13.99	12.49
16:1w5	0.00	0.00
16:1w7	1.19	0.24
18:0	4.89	3.44
18:1w9	24.67	20.96
18:1w7	0.00	0.00
18:1w5	0.00	0.00
18:2w6	29.58	50.50
18:3w6	0.03	0.02
18:3w3	19.81	10.04
18:4w3	0.09	0.03
20:0	0.28	0.28
20:1w7	0.01	0.00
20:1w9	0.44	0.27
20:2w6	0.16	0.06
20:3w9	0.01	0.00
20:3w6	0.03	0.01
20:4w6	0.20	0.02
20:3w3	0.05	0.01
20:4w3	0.07	0.01
20:5w3	0.69	0.04
22:0	0.24	0.35
22:1w9	0.03	0.03
22:4w6	0.04	0.04
22:5w6	0.04	0.00
22:5w3	0.13	0.00
24:0	0.03	0.01
22:6w3	0.96	0.11
24:1	0.04	0.02
other	1.44	0.87
sum	100.00	100.00

Supplemental Table 7. Fatty acid composition of Balanced Diet (Lab Diet, 9GU5) and Standard Rodent Chow (Mazuri, #5663). Data shown are percent of total fatty acid
